# OrthologAL: a Shiny application for quality-aware humanization of non-human pre-clinical high-dimensional gene expression data

**DOI:** 10.1093/bioinformatics/btaf311

**Published:** 2025-05-20

**Authors:** Rishika Chowdary, Robert K Suter, Matthew D’Antuono, Cynthia Gomes, Joshua Stein, Ki-Bum Lee, Jae K Lee, Nagi G Ayad

**Affiliations:** Department of Oncology, Lombardi Comprehensive Cancer Center, Georgetown University, Washington, DC 20007, United States; Department of Oncology, Lombardi Comprehensive Cancer Center, Georgetown University, Washington, DC 20007, United States; Department of Oncology, Lombardi Comprehensive Cancer Center, Georgetown University, Washington, DC 20007, United States; The Miami Project to Cure Paralysis, Department of Neurological Surgery, University of Miami Miller School of Medicine, Miami, FL 33136, United States; Department of Chemistry and Chemical Biology, Rutgers University, The State University of New Jersey, Piscataway, NJ 08854, United States; Department of Chemistry and Chemical Biology, Rutgers University, The State University of New Jersey, Piscataway, NJ 08854, United States; The Miami Project to Cure Paralysis, Department of Neurological Surgery, University of Miami Miller School of Medicine, Miami, FL 33136, United States; Department of Oncology, Lombardi Comprehensive Cancer Center, Georgetown University, Washington, DC 20007, United States

## Abstract

**Motivation:**

Single-cell and spatial transcriptomics provide unprecedented insight into diseases. Pharmacotranscriptomic approaches are powerful tools that leverage gene expression data for drug repurposing and discovery. Multiple databases attempt to connect human cellular transcriptional responses to small molecules for use in transcriptome-based drug discovery efforts. However, preclinical research often requires in vivo experiments in non-human species, which makes utilizing such valuable resources difficult. To facilitate both human orthologous conversion of non-human transcriptomes and the application of pharmacotranscriptomic databases to pre-clinical research models, we introduce OrthologAL. OrthologAL interfaces with BioMart to access different gene sets from the Ensembl database, allowing for ortholog conversion without the need for user-generated code.

**Results:**

Researchers can input their single-cell or other high-dimensional gene expression data from any species as a Seurat object, and OrthologAL will output a human ortholog-converted Seurat object for download and use. To demonstrate the utility of this application, we tested OrthologAL using single-cell, single-nuclei, and spatial transcriptomic data derived from common preclinical models, including patient-derived orthotopic xenografts of medulloblastoma, and mouse and rat models of spinal cord injury. OrthologAL can convert these data types efficiently to that of corresponding orthologs while preserving the dimensional architecture of the original non-human expression data. OrthologAL will be broadly useful for the simple conversion of Seurat objects and for applying preclinical, high-dimensional transcriptomics data to functional human-derived small molecule predictions.

**Availability and implementation:**

OrthologAL is available for download as an R package with functions to launch the Shiny GUI at https://github.com/AyadLab/OrthologAL or via Zenodo at https://doi.org/10.5281/zenodo.15225041. The medulloblastoma single-cell transcriptomics data were downloaded from the NCBI Gene Expression Omnibus with the identifier GSE129730. 10X Visium data of medulloblastoma PDX mouse models from Vo *et al.* were acquired by contacting the authors, and the raw data are available from ArrayExpress under the identifier E-MTAB-11720. The single-cell and single-nuclei transcriptomics data of rat and mouse spinal-cord injury were acquired from the Gene Expression Omnibus under the identifiers GSE213240 and GSE234774.

## 1 Introduction

Orthologous genes are paired genes in different species that originate from a common ancestor and typically have overlapping or similar functions (Gabaldón and Koonin 2013). Computational matching of orthologous genes between species in expression data is commonly facilitated by the Ensembl BioMart server (Durinck *et al.* 2005, Smedley *et al.* 2009), a free and scalable database that supports large data resources, including Ensembl and UniProt (UniProt Consortium 2023, Harrison *et al.* 2024). Single-cell and single-nuclei RNA sequencing (scRNA-seq; snRNA-seq) (Hao *et al*. 2021, 2024, Butler *et al.* 2018, Stuart *et al.* 2019) have provided significant insight into diseases and biological processes. Humanization of pre-clinical scRNA-seq and spatial transcriptomics (spatialRNA-seq) (Satija *et al.* 2015) data from other species allows for compatibility with human-derived pharmacotranscriptomic databases such as the LINCS L1000 dataset, as we and others have previously shown using bulk gene expression data (Chen *et al.* 2017, Stathias *et al.* 2018, Khalafi *et al.* 2022, Shah *et al.* 2022, Rodriguez-Blanco *et al.* 2024).

Seurat is a widely used R (R core team 2023) package specifically designed for the analysis of single-cell and spatially resolved multi-omic data. Seurat Objects in R serve as containers for single-cell and spatial transcriptomic data, and they facilitate accessible single-cell data manipulation and analysis using the Seurat library (Hao* et al.* 2021, 2024). While Ensembl's BioMart data-mining tool allows for the extraction of Ensembl data without coding knowledge and provides access to the corresponding R package, BioMaRt, the process of orthologous conversion of expression matrices in Seurat objects is not trivial for non-coders. In addition, existing approaches do not provide Seurat integration, nor do they quantify cell and pixel-level quality control (QC) metrics from high-dimensional single-cell data in the context of orthologous conversion.

To address these limitations, we created OrthologAL as a user-friendly method of performing human orthologous conversion on Seurat objects while maintaining awareness of QC metrics before and after conversion. Importantly, OrthologAL also facilitates the orthologous conversion and harmonization of input from models containing expression data from multiple species, such as that from xenograft models. Given the importance of orthologous conversion to linking pharmacotranscriptomic databases with in vivo models of human disease, we tested the utility and accessibility of OrthologAL in two use cases: medulloblastoma (MB) and spinal cord injury (SCI). Both cancer and central nervous system trauma are medical conditions that require preclinical animal models for research. Our understanding of both conditions has benefited greatly from single-cell and spatial transcriptomic profiling, given their complexity.

## 2 Features

**Figure 1.**
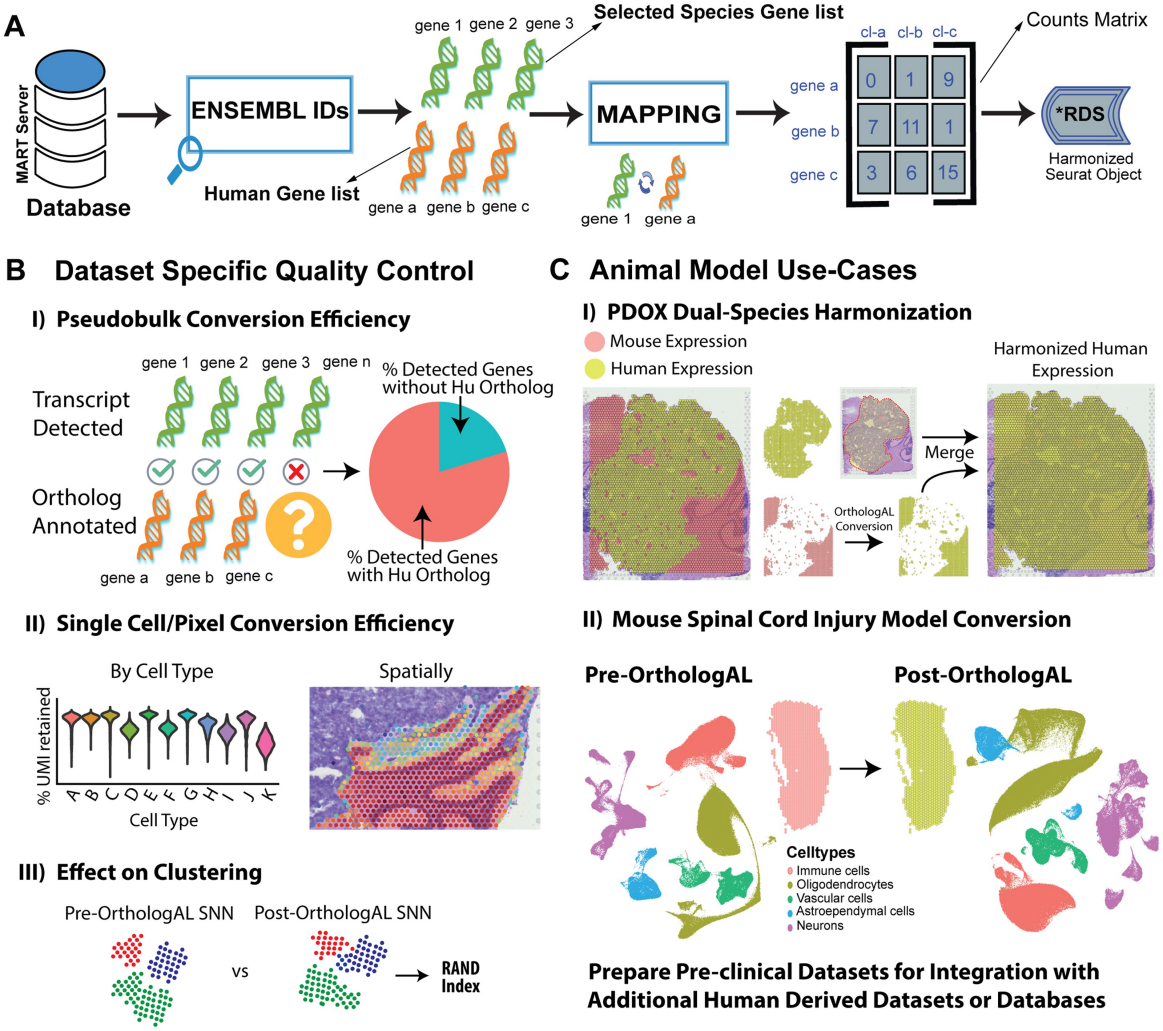
OrthologAL facilitates the conversion of non-human, single-cell, single-nuclei, or spatial transcriptomics data to that of orthologous human genes. (A) Schematic of OrthologAL conversion of non-human genes to human orthologs utilizing BioMart and Seurat object formats. (B) Dataset-specific quality controls for OrthologAL-based humanization are assessed using multiple approaches, including pseudobulk, single-cell, and pixel-level conversion efficiency. The effects of OrthologAL conversion on SNN clustering were assessed using the RAND index. (C) OrthologAL conversion was assessed in different animal models, including a mouse and human PDX tumor model, and both mouse and rat models of spinal cord injury (SCI).

OrthologAL’s Shiny (Fawcett 2018, Chang *et al.* 2024) web application allows seamless integration of BioMaRt with Seurat and provides an interface to explore QC metrics (Fig. 1). OrthologAL is available as an R package at www.github.com/AyadLab/OrthologAL. Once installed and loaded, the OrthologAL Shiny app can be launched with the single command: OrthologAL::RunOrthologAL(). OrthologAL takes a Seurat V5 object R data file (.RDS) as input and is backward-compatible with previous versions of Seurat. A drop-down menu allows the user to browse for their desired Seurat object .RDS file for easy local upload to the application. Additional drop-downs enable the user to select the species profiled in their uploaded data and choose the appropriate Seurat assay slot to be converted (e.g. “RNA” or “SCT”). By default, OrthologAL supports the orthologous conversion of mouse and rat expression data by accessing the BioMart DB. However, additional species can be processed and converted using a custom species input field. Once inputs are completed, OrthologAL maps the queried gene identifiers to their orthologs and outputs a converted Seurat Object **(**Fig. 1A, Methods 1, available as supplementary data at *Bioinformatics* online).

In contrast to other orthologous conversion tools, OrthologAL provides dataset-specific QC metrics to users (Fig. 1B). These metrics include data and visualizations for pseudobulk conversion and single-cell (or pixel-level in the case of spatial transcriptomic data) conversion efficiency. Here, we also demonstrate the evaluation of conversion efficiency by assessing downstream clustering in representative datasets. To do this, we used the RAND index to evaluate shared-nearest-neighbor (SNN) clustering concordance pre- and post-orthologous conversion. Pseudobulk and single-cell/pixel QC metrics and visualizations are provided to the user after OrthologAL-mediated conversion. Each QC metric is more extensively described in Methods 3, available as supplementary data at *Bioinformatics* online (Figs 1–5, available as supplementary data at *Bioinformatics* online).

Uniquely, OrthologAL is tailored for use with spatial and multi-species gene expression datasets (Methods 4, available as supplementary data at *Bioinformatics* online). For dual-species expression data, such as that from a patient-derived orthotopic xenograft (PDX) (Smith *et al.* 2020) in a mouse, the user can select the corresponding option from the “Run in PDX Mode” drop-down menu. In this case, OrthologAL will recognize a counts matrix that contains gene symbols or Ensembl IDs from both human and mouse transcriptomes, and it will selectively convert the expression data of mouse transcripts to their corresponding human orthologs. OrthologAL will then merge the humanized expression matrix with the original human counts present within the dataset and store this harmonized data within a new assay slot of the output Seurat object (Fig. 1C).

To benchmark the efficiency of OrthologAL and test the utility of the aforementioned QC metrics, we applied OrthologAL to single-cell or spatial transcriptomic datasets from mouse models of medulloblastoma and both mouse and rat models of SCI (Methods, Figs 1–5, available as supplementary data at *Bioinformatics* online). Both medulloblastoma and SCI require extensive pre-clinical studies and the use of animal models to develop novel therapies, rehabilitation, and treatment strategies. Analysis of single-cell and spatial transcriptomic data has significantly advanced our understanding of both conditions, and OrthologAL provides a simple, “one-click” method for orthologous conversion of such datasets. These use cases also serve to test whether OrthologAL can handle orthologous gene conversion from different species, medical conditions, and animal models across distinct technologies.

## 3 Discussion

An emerging method in drug discovery is disease signature reversal, whereby the transcriptional profile of one sample from a “disease state” is compared to that of the corresponding normal tissue (Chen *et al.* 2017, Stathias *et al.* 2018, Shah *et al.* 2022). Large pharmaco-transcriptomic datasets used in such methods are generated from human cell lines, but genetically engineered mouse models and other animal models are often necessary to study complex conditions such as cancer and injury. This makes it challenging to apply disease signature reversal and other drug discovery methods to animal model datasets. Therefore, a simple-to-use method is needed to convert non-human transcriptomic data to that of the corresponding human orthologs and facilitate direct comparison using pharmacogenomic or pharmacotranscriptomic databases.

To address this need, we developed OrthologAL. Collectively, our findings demonstrate that OrthologAL performs well in converting non-human species genes to human orthologs in two use cases across three different data types. In each species (mouse and rat), condition (medulloblastoma Figs 1 and 2, available as supplementary data at *Bioinformatics* online or SCI Figs 3 and 4, available as supplementary data at *Bioinformatics* online), and data type (scRNA-, snRNA-, and spatialRNA-seq), we evaluated pseudobulk conversion efficiency, single-cell and pixel level conversion efficiency, and the downstream effect of orthologous conversion on unsupervised clustering. Using 10X Genomics Visium data of PDX host-mouse cerebella, we also assessed the impact of OrthologAL conversion on pathway enrichment analysis using fgsea gene set co-regulation analysis (GESECA), and demonstrated a high correlation of common pathway enrichments at spot/pixel resolution (Fig. 3, available as supplementary data at *Bioinformatics* online, Supplementary File 1, available as supplementary data at *Bioinformatics* online) (Korotkevich *et al.* 2021). Likely, this can be attributed to the retention of protein-coding genes when mapping non-human genes to their corresponding orthologs using OrthologAL and BioMart (Fig. 6, available as supplementary data at *Bioinformatics* online). The OrthologAL app makes Ensembl data easily accessible to researchers by eliminating the data wrangling typically required to reformat a gene expression dataset for use with BioMart. Importantly, OrthologAL takes Seurat objects as input, a novel feature, which simplifies orthologous gene conversion for high-dimensional single-cell and spatial transcriptomic datasets. OrthologAL leverages the multi-assay Seurat object data structure to allow for parallel analysis of orthologous and original expression data. Researchers can easily install the OrthologAL package in R with minimal coding experience.

OrthologAL has multiple applications and advantages over existing options, especially in ease of use, access, and interoperability. While existing tools such as the SynGO portal and the R packages Orthogene and iGEAK do facilitate the conversion of non-human gene IDs to their human orthologs using HUGO and HGNC data, they take expression matrices or gene lists as input and do not generate QC metrics (Koopmans *et al.* 2019, Brian Schilder 2022, Choi and Ratner 2019). Distinct from these tools, OrthologAL is compatible with diverse data types, including spatial transcriptomics datasets, and has powerful, novel features such as PDX multi-species orthologous conversion and harmonization. Further, OrthologAL provides output as a downloadable Seurat object for ease of inclusion into existing pipelines. OrthologAL was developed to enhance the accessibility and FAIRness (Findable, Accessible, Interoperable, Reusable) of single-cell and spatial pharmacogenomic and pharmacotranscriptomic analyses to a broader range of researchers (Wilkinson *et al.* 2016).

With the emergence and advancement of high-dimensional single-cell and spatial technologies, tools that can convert single-cell genomic and transcriptomic data while preserving them in their main analytic environments (i.e. Seurat objects) are critical. OrthologAL produces simple-to-understand QC metrics and allows for the easy identification of genes successfully mapped to the BioMart DB. Importantly, we have shown that OrthologAL performs well on modern transcriptomic technologies such as scRNAseq, snRNA-seq, and spatial RNA-seq, including datasets that contain mixed-species gene expression, such as xenografts. We propose that this user-friendly tool will be essential in research pipelines that are repurposing and discovering new therapeutics for human disease.

## Supplementary Material

btaf311_Supplementary_Data
