# Supplementary material for: OrthologAL: a Shiny application for quality-aware humanization of non-human pre-clinical high-dimensional gene expression data
Source: Bioinformatics. 2025 May 20;41(6):btaf311. doi: 10.1093/bioinformatics/btaf311 (PMC12158155; doi:10.1093/bioinformatics/btaf311)
Supplement: btaf311_Supplementary_Data [file btaf311_supplementary_data.zip › Supplement document_noMarkup.pdf]

SUPPLEMENTARY METHODS

1. OrthologAL UI and humanized assay storage

OrthologAL takes a Seurat object R data file (.RDS) as input. Once data is uploaded to OrthologAL and the configuration parameters are set, the orthologous conversion of the Seurat object can be initiated with a single click. A status bar will appear to update the user on the progress of the conversion. During conversion, Ensembl IDs are queried against the input species codes or official gene symbols for that species if Ensembl IDs are not the primary transcript identifier within the uploaded Seurat object. This process is streamlined as data frames containing matched ortholog data for both rat and mouse genomes are stored within the OrthologAL R package to allow for rapid conversion. OrthologAL-mediated conversion will create a new, converted assay in the Seurat object with the suffix '_ortho' appended to the original assay name (i.e. 'RNA_ortho', 'Spatial_ortho', etc.). The row names of this assay are then set as the corresponding orthologous human gene symbols (HGNC (HUGO Gene Nomenclature Committee) symbols), and the associated counts are retained. The converted Seurat object will then be available to download as a .RDS file by clicking the "Download Converted Data" button.

2. BioMart Database species information

BioMart DB species	Total	Protein coding genes
Mouse Ensembl IDs	55,414	22,563
Rat Ensembl IDs	30,560	23,096
HGNC-MGI annotated genes	18,776	17,620
HGNC- RGD annotated genes	17,766	16,684

Supplementary Table 1: Table of unique mouse and rat Ensembl IDs and of how many have annotated human orthologs, respectively, in the context of both all genes and those of protein-coding genes only.

3. Single-cell and single-nuclei quality control metrics and evaluation

Using single-cell and single-nuclei datasets from both mouse and rat models of medulloblastoma or spinal cord injury, we sought to evaluate the performance of OrthologAL human ortholog conversion in terms of information retention, recognizing that valuable transcriptomic information could potentially be lost by analyzing only genes with annotated human orthologs **(Supplemental Figure S1A-B, Supplemental Figure S2A-C, Supplemental Figure S4A-B, Supplemental Figure S5A-B, Supplemental Figure S6)**.

Using the original species-aligned expression data and the data retained following ortholog mapping and conversion, we performed parallel dimensionality reduction and SNN clustering to confirm that similar clusters are identified. Comparing the resulting uniform manifold approximation projection (UMAP) and SNN clustering visually demonstrates a high degree of cluster retention after ortholog conversion **(Supplemental Figure S1D, Supplemental Figure S2D, Supplemental Figure S4D)**. For quantitative validation, we calculated the RAND index utilizing the determined cluster identities. The RAND index measures the similarity of clustering results before and after conversion with a perfect alignment giving a RAND index of 1. In all datasets tested, determined RAND indexes of clustering results were greater than 0.9. The flow of single-cell assignments to clusters before and after conversion is shown in the alluvial plots, which confirm the observed and calculated high degree of retention of cluster identity.

We also sought to quantify the data efficiency regarding the transcript biotypes retained by OrthologAL conversion. Using data from both mouse and rat models, we determined the proportions of transcripts successfully mapped to human orthologs. In both instances, the proportion of mapped transcripts significantly increases when only looking at protein-coding genes, emphasizing that the majority of genes lost to orthologous conversion are pseudogenes, alternative splicing variants, and non-coding RNAs.

Importantly, we also quantify conversion efficiency at the single cell level as a ratio of the total counts for MGI/RGD genes successfully mapped to human orthologs, to the total counts detected in the full original MGI/RGD count data for each cell

or nucleus **(Supplemental Figure S1C, Supplemental Figure S4C, Supplemental Figure S5C)**. Interrogating these single-cell data conversion efficiency ratios across identified SNN clusters or annotated cell types reveals minimal cell type or state bias in conversion. By SNN clustering, small individual clusters within each dataset did show markedly lower count retainments, likely attributed to a predominance of non-protein-coding expression characterizing these clusters. Comparing individual cell type annotations within the tabulae paralytica mouse single-nuclei RNA sequencing data, we did not observe a cell type with a mean percent count retention below 90%.

4. Spatial transcriptomics ortholog conversion and evaluation using the OrthologAL shiny App in dual-species PDX model mode

Inherently, PDX-derived scRNAseq and spatial RNA-seq datasets contain expression information for both mouse and human transcripts. Single-cell, single-nuclei, and spatial transcriptomics data can be aligned to dual-species reference transcriptomes, providing quantifications of both non-human and human expression simultaneously. In OrthologAL's PDX mode, conversion is performed on non-human transcripts, and the ortholog converted data is merged back with the original human transcript data.

Applying this approach to 10X Visium spatial gene expression data from PDX models of medulloblastoma, we assessed quality control metrics of our conversion approach. Before conversion, we identified the unique protein-coding MGI- or HGNC-annotated transcripts detected within the dataset, a critical feature performed within OrthologAL using string-matching based on the convention output by CellRanger aligning to the 10X Genomics provided dual-species reference transcriptome. Following conversion with OrthologAL, we identified the unique HGNC-annotated human orthologs matched from the original mm10 expression and performed dimensional reduction and SNN clustering of host-mouse spot data before and after conversion, showing conservation of cluster identity following ortholog conversion using the RAND index **(Supplemental Figure S2D)**. We also evaluated the percentage of gene counts retained after conversion on a spot-by-spot (pixel-by-pixel) basis. As performed for scRNAseq and snRNAseq datasets, we calculate the percentage of counts retained at each spot with mm10 detection, as the ratio of post-conversion protein-coding HGNC-converted gene counts at each spot to the total number of mm10 gene counts present for the spot before conversion **(Supplemental Figure S2B)**. Though we show a high percentage of counts are retained across most spots, we observe the loss of count data near the tumor/brain border, which we attribute to the detection of both mouse cells and invading tumor cells, and therefore relatively lower mouse transcript counts within these spots.

Within this dataset, we also determined whether pathway enrichments in the mouse brain regions of the data detected before OrthologAL conversion were similar to those detected following conversion. To do this we assayed the relative pathway enrichments in spots of the mouse cerebellum (with detected mm10 expression) pre and post-OrthologAL conversion. Using the R packages *MSigDBR* and *fgsea*, we performed gene set co-regulation analysis (GESECA) using the biological process (GOBP) pathways present in both the mouse and human databases. GESECA z-scores for pathway enrichments were extracted per spot/pixel before and after conversion. We then used the Spearman correlation to assess the similarity of the pathway enrichments determined using the original mouse cerebellar expression data (and mouse pathways DB) with those determined using OrthologAL converted data and the human pathways DB. **(Supplemental Figure S3) (Supplemental File 1)**

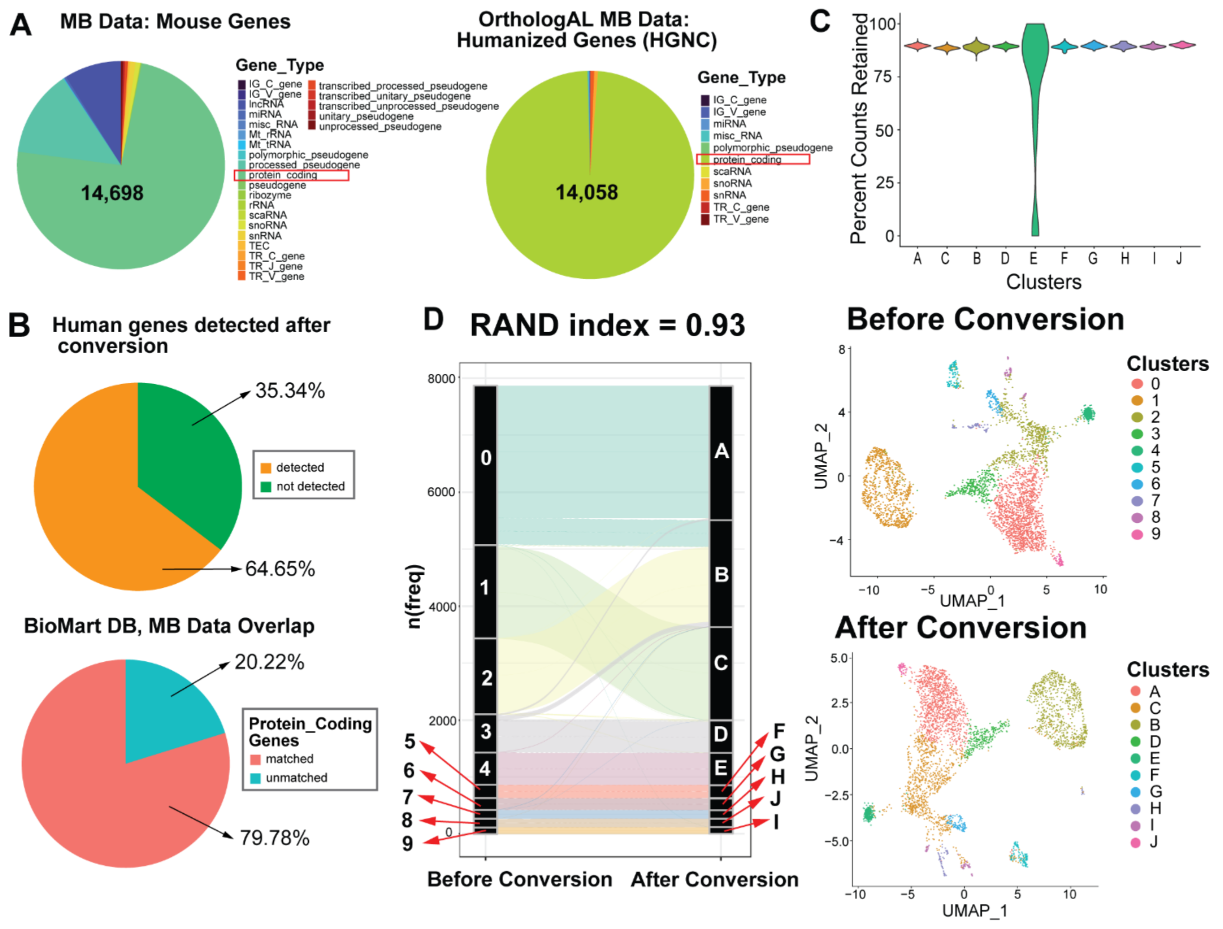

Supplemental Figure S1. Ortholog conversion via OrthologAL is a data-efficient method for the humanization of mouse single-cell RNA sequencing data A) Distribution of transcripts present in the mouse MB GEMM (C57BL/6 SmoM2-eYFP$^{loxP/loxP}$, Ocasio et al., 2019) scRNA-seq dataset (left) and distribution of genes present after orthologous conversion using OrthologAL (right). protein-coding subset is highlighted by a red box and quantified in the respective pie charts. **B)** (top) Pie chart showing the proportion of genes with counts greater than zero that were successfully converted to human orthologs. (bottom) Pie chart showing the proportion of human orthologous genes profiled by the mouse MB scRNA-seq dataset. **C)** Violin plot showing the proportion of gene counts per cell retained after orthologous conversion, stratified by post-OrthologAL SNN cluster identity. **D)** Alluvial plot showing cell assignments to SNN clusters before and after conversion. SNN clusters are labeled from 0-9 on the left (Before Conversion) and A-J on the right (After Conversion). UMAP plots before (top) and after (bottom) conversion. Cluster labels are named to match the alluvial plot.

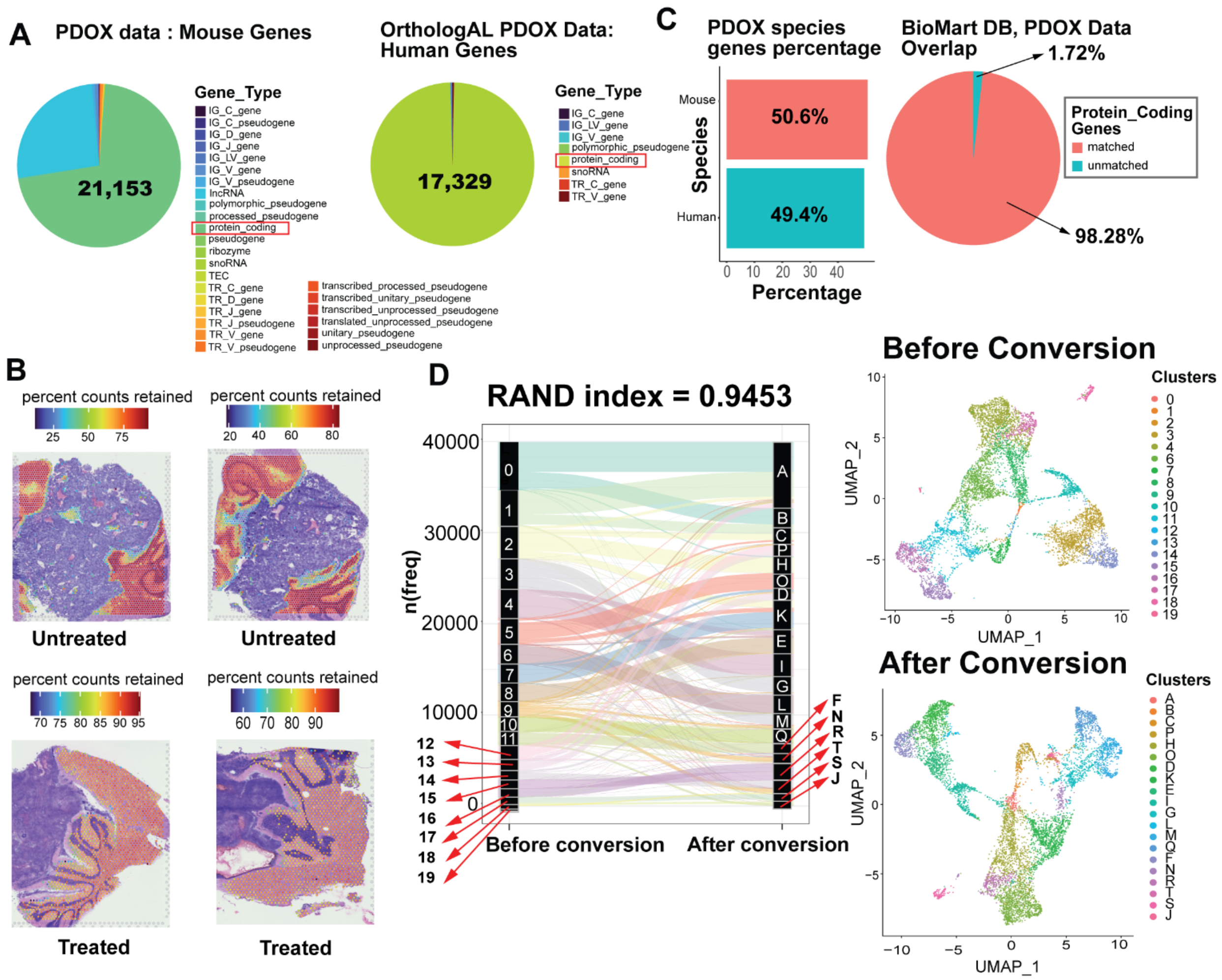

Supplemental Figure S2. OrthologAL yields a high RAND index after the conversion of non-human genes to human genes in spatial transcriptomic data. A) Distribution of genes present in the mouse MB PDOX spatialRNA-seq dataset (left) and distribution of genes present after orthologous conversion using OrthologAL (right). Protein-coding subset is highlighted by a red box and quantified in the respective pie charts. **B)** Spatial feature plots showing the percentage of data retained per spot/pixel after orthologous conversion. Percent retained is calculated for each pixel containing mouse cells as the ratio of converted counts retained in the ortholog-converted expression data relative to the total counts present for the same pixel in the original mm10 expression data. **C)** Bar plot demonstrating the proportion of mouse and human genes present in the MB PDOX spatialRNA-seq dataset (left). Pie chart showing the proportion of human orthologous genes profiled by the mouse MB PDOX dataset (right). **D)** Alluvial plot showing cell assignments to SNN clusters before and after conversion. SNN clusters are labeled from 0-19 on the left (Before Conversion) and A-T on the right (After Conversion). UMAP plots before (top) and after (bottom) conversion. Labels correspond to identities annotated in the alluvial plot in D.

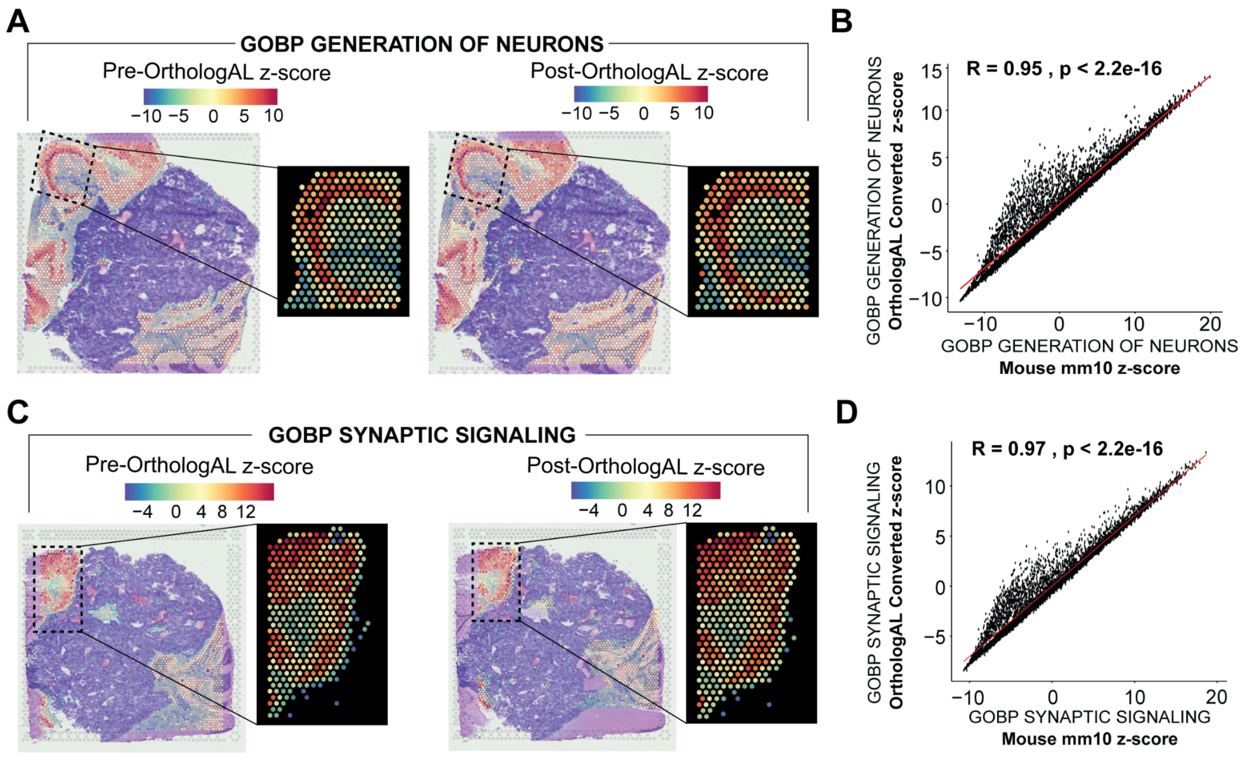

Supplemental Figure S3. OrthologAL conversion retains common pathway enrichments in cerebella of MB PDOX models. A) Spatial feature plots of a representative section showing the distribution of enrichment z-scores for the GOBP 'Generation of Neurons' pathway per spot/pixel present in mouse cerebellum (left) data and in post-OrthologAL human-ortholog converted cerebellum data (right). GOBP enrichment z-scores were calculated using GESECA and the R packages *fgsea* and *msigdbr*. **B)** Scatterplot of cerebellar spots/pixel enrichments for the GOBP 'Generation of Neurons' pathway before OrthologAL conversion using the *msigdbr* mouse DB (x-axis) and after OrthologAL conversion using the *msigdbr* human DB (y-axis). Spearman's R = 0.95, p < $2.2e^{-16}$ **C)** Spatial feature plots of a representative section showing the distribution of enrichment z-scores for the GOBP 'Synaptic Signaling' pathway per spot/pixel present in mouse cerebellum (left) data and in post-OrthologAL human-ortholog converted cerebellum data (right). GOBP enrichment z-scores were quantified using GESECA and the R packages *fgsea* and *msigdbr*. **D)** Scatterplot of cerebellar spots/pixel enrichments for the GOBP 'Synaptic Signaling' pathway before OrthologAL conversion using the *msigdbr* mouse DB (x-axis) and after OrthologAL conversion using the *msigdbr* human DB (y-axis). Spearman's R = 0.97, p < $2.2e^{-16}$. Correlations for all GOBP pathway enrichments pre- and post-OrthologAL conversion can be found in **Supplementary File 1**.

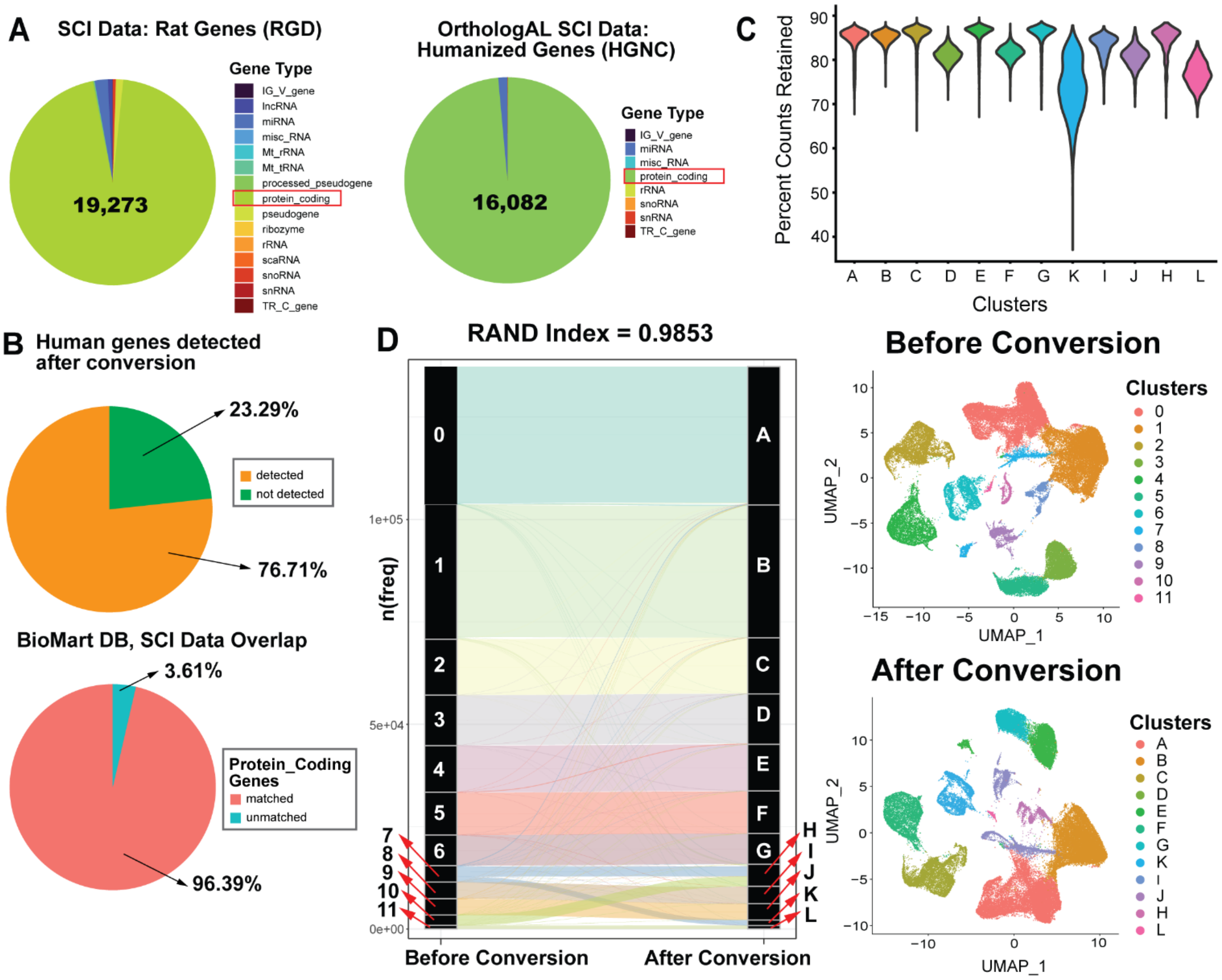

Supplemental Figure S4. OrthologAL yields a high RAND index for conversion of rat genes to human orthologs in single-cell RNA-seq data A) Distribution of transcripts present in the SCI rat scRNA-seq dataset (left) and distribution of genes present after orthologous conversion using OrthologAL (right). protein-coding subset is highlighted by a red box and quantified in the respective pie charts. **B)** Pie chart showing the proportion of genes with counts greater than zero that were successfully converted to human orthologs (top); Pie chart showing the proportion of human orthologous genes profiled by the rat SCI dataset (bottom).**C)** Violin plot showing the proportion of gene counts per cell retained after orthologous conversion, stratified by SNN cluster identity. **D)** Alluvial plot showing cell assignments to SNN clusters before and after conversion. SNN clusters are labeled from 0-11 on the left (Before Conversion) and A-L on the right (After Conversion). UMAP plots before (top) and after (bottom) conversion. Cluster labels are named to match the alluvial plot.

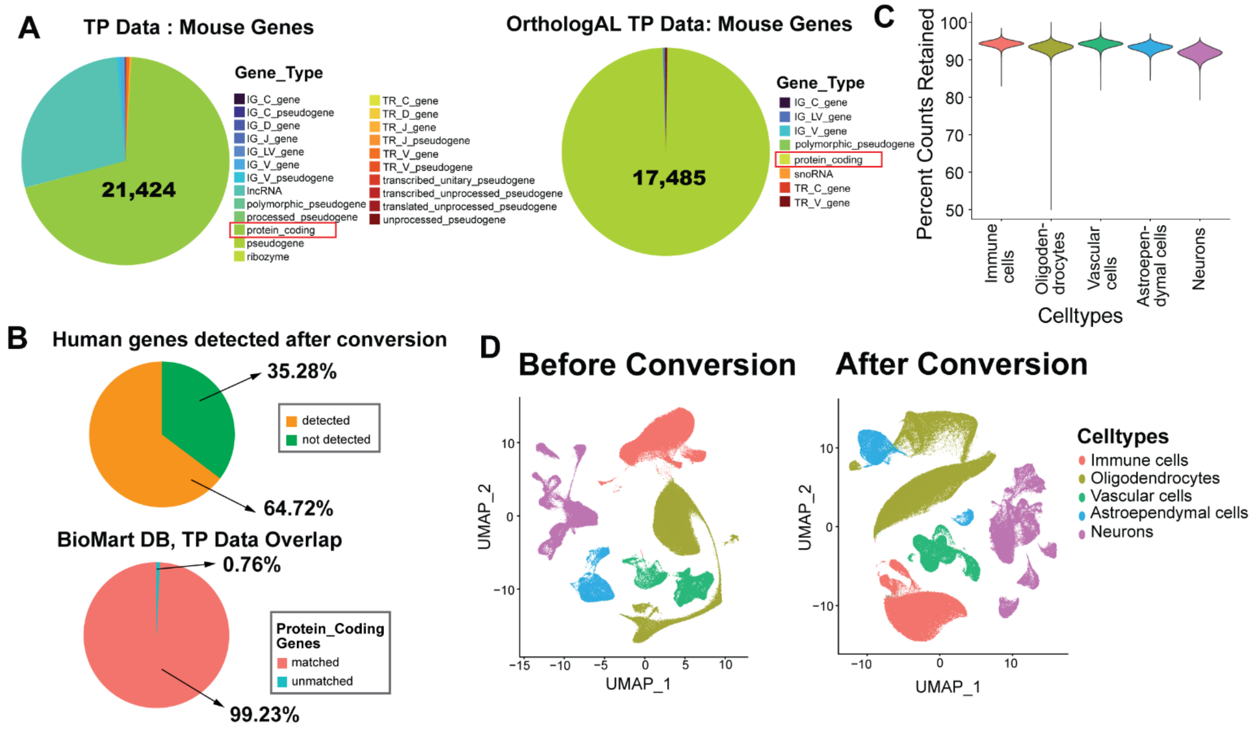

Supplemental Figure S5. OrthologAL app applied to Tabula Paralytica SCI single-nuclei data shows high concordance after conversion. A) Distribution of transcripts present in the Tabulae Paralytica dataset (left) and distribution of genes present after orthologous conversion using OrthologAL (right). Protein-coding subset is highlighted by a red box and quantified in the respective pie charts. **B)** Violin plot showing the proportion of gene counts per cell retained after orthologous conversion, stratified by cell type. **C)** Pie chart showing the proportion of genes with counts greater than zero that were successfully converted to human orthologs (top); Pie chart showing the proportion of human orthologous genes profiled by the Tabulae Paralytica dataset (bottom). **D)** UMAP plots before (left) and after (right) conversion, colored by cell type annotation.

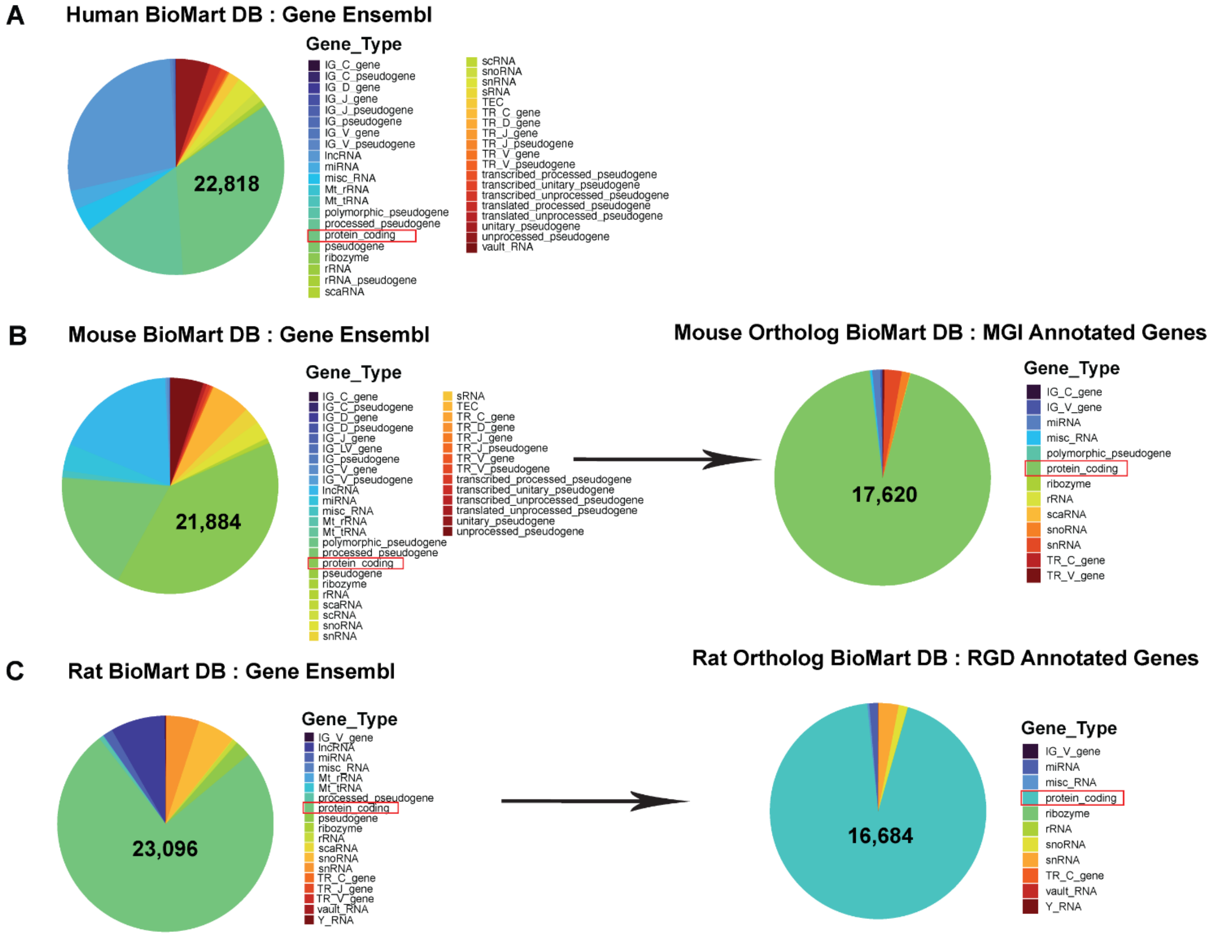

Supplemental Figure S6. OrthologAL mapping of mouse and rat genes to human orthologs preserves expression data for protein coding genes A) Distribution of human transcripts present in the BioMart DB **B)** Distribution of mouse transcripts present in the BioMart (left) and distribution of corresponding human orthologs of the whole rat database (right). **C)** Distribution of rat transcripts present in the BioMart (left) and distribution of corresponding human orthologs of the whole rat database (right).
